# Alcohol consumption and its associated factors among adolescents in a rural community in central Thailand: a mixed-methods study

**DOI:** 10.1038/s41598-022-24243-0

**Published:** 2022-11-15

**Authors:** Pichak Pramaunururut, Pijak Anuntakulnathee, Piti Wangroongsarb, Thanapat Vongchansathapat, Kullanith Romsaithong, Jareewan Rangwanich, Nuttamon Nukaeow, Poonyawee Chansaenwilai, Ploynaphat Greeviroj, Pimchanok Worawitrattanakul, Pamornwat Rojanaprapai, Veerapatra Tantisirirux, Pongpisut Thakhampaeng, Wanida Rattanasumawong, Ram Rangsin, Mathirut Mungthin, Boonsub Sakboonyarat

**Affiliations:** 1 Phramongkutklao College of Medicine, Bangkok, 10400 Thailand; 2 Department of Military and Community Medicine, Phramongkutklao College of Medicine, Bangkok, 10400 Thailand; 3 Department of Psychiatry and Neurology, Phramongkutklao Hospital, Bangkok, 10400 Thailand; 4 Department of Parasitology, Phramongkutklao College of Medicine, Bangkok, 10400 Thailand

**Keywords:** Epidemiology, Risk factors, Public health

## Abstract

Early onset of alcohol use was associated with alcohol dependence and other health problems. We aimed to identify the prevalence and factors associated with alcohol consumption among adolescents in a rural community in Thailand. A mixed-methods study was carried out in 2021 using an explanatory sequential design. The study enrolled a total of 413 adolescents. On average, young adolescents initiated alcohol consumption at age 13. The lifetime drinking prevalence among adolescents was 60.5%, while the 1-year drinking prevalence was 53.0%. The prevalence of hazardous drinking among current drinkers was 42.0%. Alcohol consumption was associated with females (adjusted prevalence ratio (APR): 1.19; 95% CI 1.01–1.41), age ≥ 16 years (APR: 1.28; 95% CI 1.09–1.50), having close friends consuming alcohol (APR: 1.75; 95% CI 1.43–2.14), night out (APR: 1.93; 95% CI 1.53–2.45), being a current smoker (APR: 1.39; 95% CI 1.15–1.69), and having relationship (with boyfriend/girlfriend) problems (APR: 1.18; 95% CI 1.01–1.38). Qualitative data demonstrated that individual and environmental factors, including friends, family, social media use, and alcohol accessibility, affect alcohol use in this population. Therefore, effective strategies should be implemented across multiple levels of the socio-ecological model simultaneously to alleviate alcohol consumption and attenuate its complications.

## Introduction

Alcohol consumption is associated with various health issues, such as maternal and child health, infectious diseases, noncommunicable diseases, mental health, and injuries^1^.

The National Statistical Office (NSO) in Thailand reported that, during 2003–2015, the prevalence of current alcohol consumption among adults aged at least 15 years consistently ranged from 32.7 to 34.0% and then reduced slightly to 28.4% in 2017^2^. Current alcohol consumption among Thai males (47.5%) is more likely to be higher than that among females (10.6%). As regards the residential area, the prevalence of current alcohol consumption was 29.3% and 27.4% outside and within the municipal area, respectively^2^.

Early onset of alcohol consumption was associated with a higher risk of heavy alcohol drinking^3^, substance use^4^, and poorer performance in psychomotor speed and visual attention^5^. In Thailand, national policies and interventions to solve this issue have been implemented, including the national legal minimum age for on-/off-premises sales of alcoholic beverages, restrictions for on-/off-premises sales of alcoholic beverages (hours, days/places, and density), and national level government support for community action^1^. Unfortunately, a continuous increase in the incidence of early alcohol consumption (before the age of 20 years) has been observed, which ranged from 8.9% in 2007 to 10.9% in 2017^6,7^.

The Global School-Based Student Health survey illustrated that the prevalence of current alcohol use among Thai adolescents aged 13–17 years increased from 14.8% in 2008 to 22.2% in 2015^8^. Furthermore, the Global Status Report on Alcohol and Health 2018 presented that alcohol consumption among Thai adolescents was top-ranking in Southeast Asia^1^. Recent studies revealed several factors associated with current alcohol consumption among adolescents, such as family problems, direct observation of friends’ drinking, having parents store alcohol at home, and exposure to alcohol advertising. In addition, risky sexual and suicidal behaviors were associated with alcohol use^9,10^.

Although national data include information about alcohol consumption in Thailand^2^, sufficient information about the socio-ecological context associated with alcohol consumption among adolescents in a particular community is still limited. A few studies investigating alcohol consumption among adolescents were conducted in urban and suburban areas, presenting an estimated prevalence of current alcohol consumption ranging from 10.4 to 18.6%^9–11^. Nowadays, one-half of the Thai population still resides in rural areas where healthcare provider characteristics, health literacy, and socioeconomic contexts may differ from those in urban areas^12^. Recently, a related quantitative study about the substance abuse situation in a remote rural community, Chachoengsao Province, detected that 36.8% of adolescents reported being a current alcohol drinker and indicated that alcohol drinking was associated with substance abuse in the rural community^4^, which was considered a major health issue in the Thai rural community. Unfortunately, information regarding risk factors for alcohol consumption among adolescents in this remote rural area was not uncovered.

The present study aimed to identify the prevalence and factors associated with alcohol consumption among adolescents in a remote rural area using quantitative methods. Moreover, a qualitative study will explore the socio-ecological factors affecting alcohol consumption, including family members and friends, store and accessibility, and social and environmental factors. If factors associated with alcohol consumption among adolescents are explored, appropriate strategies and practical interventions may be implemented in this population to resolve this issue in the future.

## Methods

### Study design and subjects

The current study was performed in a remote rural area in Thakradan Subdistrict, Sanam Chai Khet District, at the border area of Chachoengsao Province, central Thailand, 160 km east of Bangkok. Due to their location in the border area of the province, a total of five villages (from 23 villages) in the Thakradan Subdistrict were selected to conduct the study. Furthermore, since 2002, these five villages have been known as the Na-Yao community, where Phramongkutklao College of Medicine established the teaching community for undertaking community-based research. In 2018, a related study in this community revealed that alcohol use among adolescents in this rural area is relatively high (36.8%) and also related to substance use. Nevertheless, information on factors associated with alcohol use was unavailable. Therefore, we aimed to employ a mixed-methods study using explanatory sequential design in this area to explore the magnitude of alcohol use and also determine socio-ecological factors affecting alcohol use among adolescents in this rural context. Regarding the explanatory sequential mixed method, we collected quantitative data about adolescents and then collected qualitative data through the use of in-depth interviews and focus group discussions among adolescents, teachers, parents, and adults in the community to help explain the quantitative results.

#### Quantitative part

We conducted a cross-sectional quantitative study to identify the prevalence and factors associated with alcohol consumption among adolescents in March 2021. The minimal calculated sample size of 341 was determined according to the 2018 Global Status Report on Alcohol and Health of Thai adolescents^1,13^. We expected that 25% of available adolescents would be unable to participate. Thus, 427 were finally estimated to mitigate the effect of possible losses. We carried out a quantitative method at school *A*, which is a government high school providing education for adolescents aged 13 to 18 years residing in this community. The exclusion criteria included individuals who did not provide informed consent or could not answer the questionnaire. Four hundred twenty-five adolescents in school *A* were invited to participate in the study, and 413 individuals agreed to participate.

#### Qualitative part

We also performed a qualitative study to investigate the socio-ecological factors that affect alcohol consumption among adolescents. At high school *A*, the investigator invited adolescents, teachers, parents, and adults in the community to participate in the qualitative study. Moreover, advertising posters were provided at school to encourage target participants to participate in the study. Purposive sampling was also utilized for selecting six adolescents, three teachers, six parents, and adults in this community for in-depth interviews. Forty-nine adolescents, seven teachers, and five parents and adults in the community were purposively selected for focus group discussions. Nine focus groups (FGs) included adolescents, teachers, parents, and adults in the community. The first FG to seventh FG (n = 7/group) consisted of adolescents. The eighth FG (n = 7) comprised teachers from high school, whereas the ninth FG (n = 5) comprised parents of adolescents and adults. Forty-nine adolescents who participated in the quantitative study also participated in the FGs.

### Data collection

#### Quantitative part

After permission was received from the director of school *A*, the investigators invited adolescents in school *A* to participate in the quantitative part of the study. Additionally, an advertising poster of the present study was provided in front of the classroom to encourage them to participate in the study. Furthermore, information sheets, objectives, and study methods were provided to the subjects. Informed consent was obtained prior to the research. During the study, self-reported questionnaires were utilized to obtain essential information from participants within 20 min. The questionnaires were self-administered and delivered in an envelope. To de-identify, a unique identification number was used instead of the names and identities of the volunteers. An adolescent could decide not to participate in the study. This decision does not affect any dimensions of adolescents, such as education and health care.

Standardized questionnaires developed by the investigators for the current study were divided into three parts as follows: demographic characteristics, associated factors, and history of alcohol consumption. They included information regarding demographics, including sex, age, parental status, religion, educational level, and grade point average (GPA). The characteristics of alcohol consumption were collected through the use of standardized questionnaires asking about lifetime prevalence and the last 12-month prevalence of alcohol consumption, age at initial alcohol consumption, and categories of alcoholic beverages including beer, whiskey, white liquor, and wort. Lifetime alcohol consumption and the last 12-month prevalence of alcohol consumption were defined based on the data obtained from the responses to the following questions: (1) “Have you ever consumed any alcohol such as beer, wine, spirits, and wort?” and (2) “Have you consumed any alcohol within the past 12 months?”, respectively^14^. Among the participants consuming alcohol in the last 12 months, hazardous drinking was assessed using the Alcohol Use Disorders Identification Test (AUDIT) (score ≥ 8)^15^.

#### Qualitative part

The investigators (BS, TV, PT, and VT), who were the interviewers, were trained at the Phramongkutklao College of Medicine before conducting the qualitative study. The interviews were done in the meeting room of high school *A* in the community. Informed consent was obtained before the research. Two researchers (BS and TV) conducted in-depth interviews and took notes on non-verbal communication. Interviews of the nine FGs were facilitated by two investigators (BS and TV). In addition, notes on non-verbal communication and other notable items were taken by two researchers (PT and VT). All investigators wrote additional notes after the interviews of the FGs. The interviews were carried out with questions and probes for further questioning, covering questions on the factors influencing alcohol consumption among adolescents, how adolescents access alcohol, and existing interventions in the community (Supplementary File 1). The data were collected continuously until the contents were saturated. The conversations were taped using a voice recorder and transcribed into text. Two investigators (BS and TV) reviewed the transcription to check the errors before performing an analysis.

### Statistical analysis

#### Quantitative part

Data were analyzed using StataCorp, 2021, *Stata Statistical Software: Release 17*, College Station, TX, USA, StataCorp LLC. Demographic characteristics were determined through the use of descriptive statistics. The age of participants was categorized into two groups (< 16 and $\ge$ 16 years) regarding median age. According to the NSO report in Thailand, the age at initiation of alcohol consumption was categorized into two groups: < 15 and $\ge$ 15 years. The frequency distribution of categorical variables by strata was compared using the *Chi*-square test, while continuous data were compared using Student’s* t*-test. Through descriptive statistics, lifetime prevalence and last 12-month prevalence of alcohol consumption were reported as a percentage with a 95% confidence interval (CI). The univariable analysis was utilized to identify the associated factors of the last 12 months of alcohol consumption. Multivariable analysis was performed. After running a logit model, the adjrr (margin) command was used to calculate the adjusted prevalence ratio (APR), which was presented with a corresponding 95% CI. A two-sided *p*-value less than 0.05 was considered statistically significant.

#### Qualitative part

The qualitative study employed a thematic analysis. Analytic rigor in the qualitative analysis was ensured through investigator triangulation^16^. Transcripts were compared to the investigators' notes taken during the in-depth interviews and focus groups (BS and TV). The text-based data transcribed from the conversation were sorted and coded. Inductive and deductive coding were used, and analytic categories and themes were then developed. Initial coding and themes (by BS and TV) were checked by the other investigators (PP and VT)^17^. The quotations below best represent the range of ideas voiced around key themes.

### Ethics consideration

The current study was reviewed and approved by the Institutional Review Board, Royal Thai Army Medical Department according to international guidelines including the Declaration of Helsinki, the Belmont Report, CIOMS Guidelines, and the International Conference on Harmonization of Technical Requirements for Registration of Pharmaceuticals for Human Use-Good Clinical Practice (ICH-GCP) (approval number R190q/63). Written informed consent was obtained from the participants according to the WMA Declaration of Helsinki Ethics Principles for medical research involving human subjects.

## Results

### Quantitative study

#### Demographic characteristics of participants

In a rural community, a total of 425 adolescents were invited to the present study, among which 413 (97.2%) responded to participate in the study. The demographic data of these participants are shown in Table 1. Among them, 250 (60.5%) were females. The average age of the participants was 15.5 ± 1.6 years (ranging from 13 to 18). The adolescents were in grades 7 to 12 and vocational training. One-half of the participants (49.9%) lived with both a father and a mother.

**Table 1:** Demographic characteristic of participants (n = 413).

Characteristics	n (%)
**Sex**	
Female	250 (60.5)
Male	163 (39.5)
**Age (years)**	
< 16	199 (48.2)
≥ 16	214 (51.8)
Mean ± SD	15.5 ± 1.6
Median (min–max)	16.0 (13.0–18.0)
**Religions**	
Buddhist	405 (98.1)
Christ	8 (1.9)
**Parental status**	
Couple	230 (62.2)
Divorced/separated	10 (2.7)
Widowed	130 (35.1)
**Living with in latest 12 months**	
Both father and mother	205 (49.9)
Relative(s)	126 (30.7)
Mother	59 (14.4)
Father	14 (3.4)
Alone	5 (1.2)
Friend(s)	2 (0.5)
**Place where you live in latest 12 months**	
Home	401 (97.6)
Dormitory	7 (1.7)
Friend’s house	3 (0.7)
**Education attainment**	
Grade 7	73 (17.7)
Grade 8	66 (16.0)
Grade 9	69 (16.7)
Grade 10	92 (22.3)
Grade 11	65 (15.7)
Grade 12	47 (11.4)
Vocational training	1 (0.2)
**Grade point average (latest semester)**	
< 2.50	25 (14.3)
2.50–3.49	104 (59.4)
3.50–4.00	46 (26.2)
Mean ± SD	3.1 ± 0.6
Median (min–max)	3.2 (1.0–4.0)
**Having girlfriend/boyfriend**	
No	306 (76.9)
Yes	92 (23.1)
**Pocket money (Baht/month)**	
< 1200	102 (25.8)
≥ 1200	294 (74.2)
Mean ± SD	1368.2 ± 718.5
Median (min–max)	1200.0 (600.0–6000.0)

*SD* standard deviation.

#### Prevalence of alcohol consumption among adolescents in a Thai rural community

The characteristics of alcohol consumption among adolescents are presented in Table 2. The average age at initial alcohol consumption was 13.9 ± 1.8 years (ranging from 10 to 18); 59.6% of drinkers started drinking at age less than 15. The lifetime drinking prevalence among adolescents was 60.5% (95% CI 55.6–65.3%). The overall one-year drinking prevalence was 53.0% (95% CI 48.1–57.9%) and was comparable between 52.1% (95% CI 44.2–60.0%) among males and 53.6% (95% CI 47.2–59.9%) among females (*p*-value = 0.772). Hazardous drinking determined by AUDIT score among current drinkers was 42.0, 41.2, and 42.5% among total, male, and female participants (*p*-value = 0.842), respectively. Beer and whiskey were the most prevalent beverages among adolescents accounting for 59.3 and 20.0%, respectively.

**Table 2:** Characteristic of alcohol consumption among adolescents in a rural community, central, Thailand.

Characteristics	Overall	Male	Female	*p*-value
	n (%)	n (%)	n (%)	
**Age at initiation of alcohol consumption**				0.276^†^
Total, n	213	78	135	
Mean ± SD	13.9 ± 1.8	13.8 ± 2.0	14.0 ± 1.7	
Median (min–max)	14 (10–18)	14 (10–18)	14 (10–18)	
< 15 years	127 (59.6)	46 (59.0)	81 (60.0)	0.883^§^
$\ge$ 15 years	86 (40.4)	32 (41.0)	54 (40.0)	
**Lifetime prevalence**				0.891^§^
Total, n	413	163	250	
Prevalence	250 (60.5)	98 (60.1)	152 (60.8)	
95% CI	55.6–65.3	52.2–67.7	54.5–66.9	
**Last 12-month prevalence**				0.772^§^
Total, n	413	163	250	
Prevalence	219 (53.0)	85 (52.1)	134 (53.6)	
95% CI	48.1–57.9	44.2–60.0	47.2–59.9	
**Hazardous and harmful alcohol use by AUDIT score**				0.842^§^
Simple drinker (AUDIT < 8)	127 (58.0)	50 (58.8)	77 (57.5)	
Hazardous drinker (AUDIT ≥ 8)	92 (42.0)	35 (41.2)	57 (42.5)	
**Categories of alcoholic beverages**				0.262^§^
Total, n	204	81	123	
Beer	121 (59.3)	54 (66.7)	67 (54.5)	
Whiskey	41 (20.0)	11 (13.6)	30 (24.4)	
White liquor	27 (13.2)	10 (12.3)	17 (13.8)	
Wort	3 (1.5)	2 (2.5)	1 (0.8)	
Others	12 (5.9)	4 (4.9)	8 (6.6)	

*SD* standard deviation.

^†^*t*-test (male vs female).

^§^*Chi*-square test (male vs female).

#### Associated factors of alcohol consumption among adolescents in a Thai rural community

Univariable and multivariable analyses were carried out to identify the associated factors of alcohol consumption within 12 months (Tables 3, 4). After adjusting for potential confounders, the independent factors associated with alcohol consumption within 12 months included females (APR: 1.19; 95% CI 1.01–1.41), age ≥ 16 years (APR: 1.28; 95% CI 1.09–1.50), having close friends consuming alcohol (APR: 1.75; 95% CI 1.43–2.14), night out (APR: 1.93; 95% CI 1.53–2.45), being a current smoker (APR: 1.39; 95% CI 1.15–1.69), and having relationship (with boyfriend/girlfriend) problems (APR: 1.18; 95% CI 1.01–1.38).

**Table 3:** Univariable analysis for factors associated with last 12-month alcohol consumption among adolescents in a rural community, central, Thailand.

Factors	Alcohol consumption within 12 months		Unadjusted PR (95% CI)	*p*-value
	Yes, n (%)	No, n (%)		
**Sex**				
Male	85 (52.1)	78 (47.9)	1	
Female	134 (53.6)	116 (46.4)	1.03 (0.85–1.24)	0.773
**Age (years)**				
mean ± SD	15.9 ± 1.6	15.0 ± 1.6	1.42 (1.24–1.61)	< 0.001
< 16	79 (39.7)	120 (60.3)	1	
≥ 16	140 (65.4)	74 (34.6)	1.65 (1.35–2.01)	< 0.001
**Pocket money (Baht/month)**				
< 1200	35 (34.3)	67 (65.7)	1	
≥ 1200	176 (59.9)	118 (40.1)	1.74 (1.31–2.32)	< 0.001
**Grade point average (latest semester)**				
≥ 2.50	69 (46.0)	81 (54.0)	1	
< 2.50	15 (60.0)	10 (40.0)	1.30 (0.91–1.88)	0.153
**Having girlfriend/boyfriend**				
No	146 (47.7)	160 (52.3)	1	
Yes	63 (68.5)	29 (31.5)	1.44 (1.20–1.72)	< 0.001
**Parental status**				
Couple	121 (52.6)	109 (47.4)	1	
Widowed	5 (50.0)	5 (50.0)	0.95 (0.51–1.79)	0.872
Divorced/Separated	69 (53.1)	61 (46.9)	1.01 (0.82–1.24)	0.932
**Current smoke**				
No	161 (47.2)	180 (52.8)	1	
Yes	55 (82.1)	12 (17.9)	1.74 (1.48–2.04)	< 0.001
**Like a challenge**				
No	90 (47.9)	98 (52.1)	1	
Yes	128 (57.4)	95 (42.6)	1.20 (0.99–1.45)	< 0.001
**History of destroying public goods**				
No	174 (51.9)	161 (48.1)	1	
Yes	44 (58.7)	31 (41.3)	1.13 (0.91–1.40)	0.292
**Social media use**				
No	4 (44.4)	5 (55.6)	1	
Yes	214 (53.6)	185 (46.4)	1.21 (0.58–2.52)	0.587
**Time spent on social media use**				
< 6 h	41 (36.3)	72 (63.7)	1	
≥ 6 h	109 (59.9)	73 (40.1)	1.65 (1.26–2.17)	< 0.001
**History of dropping out from school**				
No	204 (52.2)	187 (47.8)	1	
Yes	11 (73.3)	4 (26.7)	1.41 (1.02–1.93)	0.107
**Having close friend(s)**				
No	13 (43.3)	17 (56.7)	1	
Yes	204 (53.7)	176 (46.3)	1.24 (0.81–1.88)	0.274
**Having close friend consuming alcohol**				
No	61 (28.4)	154 (71.6)	1	
Yes	144 (80.9)	34 (19.1)	2.85 (2.28–3.57)	< 0.001
**Insomnia in latest 12 months**				
No	101 (47.4)	112 (52.6)	1	
Yes	113 (59.2)	78 (40.8)	1.25 (1.04–1.50)	< 0.018
**Feeling lonely in latest 12 months**				
No	105 (47.7)	115 (52.3)	1	
Yes	113 (59.2)	78 (40.8)	1.24 (1.03–1.49)	0.021
**Suicidal idea in latest 12 months**				
No	178 (51.3)	169 (48.7)	1	
Yes	40 (62.5)	24 (37.5)	1.22 (0.98–1.51)	0.099
**Suicidal attempt in latest 12 months**				
No	195 (51.5)	184 (48.5)	1	
Yes	23 (71.9)	9 (28.1)	1.40 (1.10–1.77)	0.026
**School truancy in latest 12 months**				
No	86 (39.1)	134 (60.9)	1	
Yes	132 (69.1)	59 (30.9)	1.77 (1.46–2.14)	< 0.001
**Parental empathy in latest 12 months**				
No	46 (64.8)	25 (35.2)	1	
Yes	170 (50.4)	167 (49.6)	0.78 (0.64–0.95)	0.029
**Being bullied in latest 12 months**				
No	111 (49.1)	115 (50.9)	1	
Yes	104 (57.8)	76 (42.2)	1.18 (0.98–1.41)	0.083
**Getting into a fight in latest 12 months**				
No	170 (51.5)	160 (48.5)	1	
Yes	48 (59.3)	33 (40.7)	1.15 (0.93–1.42)	0.211
**Night shift work in latest 12 months**				
No	195 (52.3)	178 (47.7)	1	
Yes	13 (65.0)	7 (35.0)	1.24 (0.89–1.74)	0.267
**Night out in latest 12 months**				
No	42 (24.3)	131 (75.7)	1	
Yes	174 (75.0)	58 (25.0)	3.09 (2.35–4.06)	< 0.001
**Having relationship (with boyfriend/girlfriend) problem in latest 12 months**				
No	105 (42.0)	145 (58.0)	1	
Yes	114 (69.9)	49 (30.1)	1.67 (1.39–1.99)	< 0.001
**Having study problem in latest 12 months**				
No	124 (48.8)	130 (51.2)	1	
Yes	95 (59.7)	64 (40.3)	1.22 (1.02–1.46)	0.030
**Having friend problem in latest 12 months**				
No	118 (48.4)	126 (51.6)	1	
Yes	100 (59.9)	67 (40.1)	1.24 (1.03–1.48)	0.022
**Having financial problem in latest 12 months**				
No	97 (43.9)	124 (56.1)	1	
Yes	122 (63.9)	69 (36.1)	1.46 (1.21–1.75)	< 0.001
**Having family problem in latest 12 months**				
No	135 (48.6)	143 (51.4)	1	
Yes	84 (62.2)	51 (37.8)	1.28 (1.07–1.53)	0.009
**Attending religious activities in latest 12 months**				
No	72 (54.1)	61 (45.9)	1	
Yes	144 (52.6)	130 (47.4)	0.97 (0.80–1.18)	0.764
**Plan for university**				
No	14 (66.7)	7 (33.3)	1	
May be	128 (53.1)	113 (46.9)	0.80 (0.58–1.10)	0.232
Yes	75 (51.4)	71 (48.6)	0.77 (0.55–1.08)	0.189

*PR* prevalence ratio, *95% CI* 95% confidence interval, *SD* standard deviation.

**Table 4:** Multivariable analysis for factors associated with last 12-month alcohol consumption among adolescents in a rural community, central, Thailand.

Factors	Adjusted prevalence ratio	95% CI	*p*-value
**Sex**			
Male	1		
Female	1.19	1.01–1.41	0.040
**Age (years)**			
< 16	1		
≥ 16	1.28	1.09–1.50	0.003
**Having close friend consuming alcohol**			
No	1		
Yes	1.75	1.43–2.14	< 0.001
**Night out in latest 12 months**			
No	1		
Yes	1.93	1.53–2.45	< 0.001
**Current smoke**			
No	1		
Yes	1.39	1.15–1.69	< 0.001
**Having relationship (with boyfriend/girlfriend) problem in latest 12 months**			
No	1		
Yes	1.18	1.01–1.38	0.037

Adjusting for sex, age, having close friend consuming alcohol, night out, current smoke, having relationship (with boyfriend/girlfriend) problem.

*CI* confidence interval.

### Qualitative study

#### Demographic characteristics of participants

For in-depth interviews, data were collected from a total of 15 participants, including 6 adolescents, 6 parents and adults in the community, and 3 teachers. For focus group discussions, data were gathered from a total of 61 participants, including 49 adolescents (aged 13 to 18 years), 5 parents and adults in the community (aged 19 to 63 years), and 7 teachers (aged 44 to 48 years). The information from in-depth interviews and focus group discussions can be grouped into more factors and interventions.

#### Knowledge of disadvantages of alcohol consumption among adolescents

Adolescents were aware of the short-term negative effects of alcohol consumption on mental and physical health, for instance, encouraging aggression, decreasing the level of consciousness, and facilitating road traffic accidents. However, they did not realize the long-term health risks of excessive alcohol use, including chronic liver disease and gastrointestinal hemorrhage. A 21-year-old man, working freelance, said, “If you know the disadvantage, you will drink less. It can be reduced anyway”. A 13-year-old talked about the disadvantages of consuming alcohol, “create a bad mood, more aggressive and threatening behaviors to other people. Some people even hurt family members. I have seen people whose motorcycles collide because they drove drunk”. From the inquiries of the participants, regarding the pros and cons of consuming alcohol, many commented that they did not have knowledge regarding the effects of alcohol on the body, like health aspects including liver disease and mental health.

#### Personal factors affecting alcohol consumption among adolescents

Curiosity and ignorance of individuals provoke the urge for trial and entertainment, especially during important festivals such as the Songkran water festival and New Year’s Eve. An adolescent stated, “I wanted to try drinking so I asked my dad”. However, the parents believed that adolescents with good judgment tended to drink lightly. As they disclosed, “If the adolescent can think for himself/herself, then abstinence may be possible, but if he/she drinks and goes partying at night and has a group of friends who do the same, then the chances of quitting are quite low”. “Teens drink according to their judgment and will regarding their knowledge of the pros and cons of alcohol”.

#### Friends affecting alcohol consumption among adolescents

Most adolescents thought that their initial alcohol consumption was due to an invitation from their friends or seniors. A 19-year-old maintained, “I was in grade 7 in a boarding school and often visited my senior’s flat to hang out with my friends”. Another 13-year-old girl commented, “a friend tricked her into drinking by putting alcohol in the bottle and affirmed that it was punch. Friends often hang out in groups to drink in private areas”. “Her friends invite her on weekends to a friend’s house or a restaurant that sells alcohol”.

Teachers pointed out that adolescents without sufficient care in the family would be persuaded by their friends to consume alcohol. A 48-year-old teacher stated, “He saw an 18-year-old teen drink after work with his/her friend during a festival because his/her friend invited him. Some kids do not reside with their parents and live with their grandparents who may lack sufficient care”. Parents believed that friends were a major facilitator of alcohol consumption among adolescents. One parent group mentioned, “Friends are the main contributors to consuming alcohol. In the curiosity of the adolescents, they thought that everyone who drinks seems to enjoy themselves and a person who gets drunk will be seen as a funny person, which was inaccurate”.

#### Family factors affecting alcohol consumption among adolescents

Some adolescents faced family disharmony including the separation of their parents, while others had grandparents and relatives as their main guardians. Therefore, adolescents were not taken care of and expressed inappropriate behaviors including alcohol consumption. A 60-year-old uncle reported, “Most of the parents did not know that the children drank because the children drank outside of the home”. The parents commented, “the influence of family members consuming alcohol facilitates the children to drink. Another contributing factor is problems in the family institution, such as separation”.

Furthermore, one teacher believed that smoking may initiate alcohol consumption. Alcohol consumption among adolescents resulted from imitating family members who smoke, which could lead them to consume alcohol later. A 44-year-old teacher revealed, “When children begin to show up to be at risk, the first thing you can find is that the cigarette may not be a real cigarette. Sometimes a paper substitute can be a simulated behavior. Cigarettes are easier to find than alcohol because parents and other older members had them at home. Children could pick them up easily. Then they may arrange an appointment with friends to buy liquor and drink as the next step”. On the other hand, adolescents believed that alcohol use in the family would not influence teenagers to drink alcohol accordingly.

#### Social and environmental factors affecting alcohol consumption among adolescents

Children gain various experiences in the society and environment in which they grow up. Thus, these factors will greatly affect their behaviors when they become adolescents. A 44-year-old teacher indicated, “Environment and society have a great effect on teenager experience. The new generation is growing by changing their lifestyle to create their own identity. After an adolescent receives information, they cannot distinguish right or wrong, and the bad things are easier than the good”. Another teacher suggested, “I think children are used to behaving well, but they have changed over time and by their friends; however, this group can repent and be guided correctly. On the other hand, if the family did not help from the beginning, for example, some children are born unwilling or do not feel what the word “love” is, this group will be a little tricky. It means that it is his/her way of life which is very difficult to change”.

#### Advertising and cyber society factors affecting alcohol consumption among adolescents

Teachers and parents thought that adolescents easily access advertising and cyber society, in which the evolution of technology is present. Therefore, adolescents could watch any story including alcohol-drinking behavior on social media which may influence their behavior in the future. A 44-year-old teacher explained, “Lacking media literacy and using too much technology on the mobile phone, celebrity, games, or anything easy to access and fast, these things are shaping and creating the new generation without an appropriate evaluation”. A group of parents responded, “Some children watch the drinking behavior from the media and think it is normal”.

#### Financial factors affecting alcohol consumption among adolescents

Many people thought that drinking alcohol could solve financial problems, for example, and that stress problems and anxiety would be eliminated by drinking. Unfortunately, it placed additional financial burden on them because they had to find more money to buy alcohol. A 21-year-old man expressed, “I started smoking and drinking for the first time in grade 6 because of stress from the fact that people had more money to go to school than me. He added that money to buy alcohol comes in various ways, saying there are many ways to get money—from parents, stealing, and doing illegal things. Having voiced that, what can be converted into money, for example, to steal a phone”.

#### Access to alcohol in the rural community

Young people could easily buy alcohol in the shops. Sellers did not have restrictions about selling liquor to children under 20 years, so teenagers could buy and drink by themselves. They did not ask about the age of the children when buying alcohol. If there were children to buy alcohol, they tended to understand that their parents asked them to buy. In addition, alcohol could be bought in the area around the school. A 13-year-old boy alleged, “easy access to the shops nearby school. In some stores, children can buy by themselves”. A 14-year-old girl acknowledged, “You can buy liquor from a store without any limited age. The merchant thinks they buy it for adults”. A 53-year-old woman replied, “The liquor store sells to even children under 18, not strict. Sometimes the child claims to buy it for his parents, and the shop sells it”. A 44-year-old teacher said, “Access to alcohol, I think, is not difficult. Teenagers can find all these things everywhere. The word shame should never exist in the minds of these teenagers”.

#### Alcohol consumption intervention in a Thai rural community

The existing intervention included education from schools, health services, or policies for controlling alcohol access within the school-based program. A 15-year-old boy asserted, “The school has a rule that bringing alcohol to school is not allowed by random examination”. A 13-year-old girl insisted, “Public health service used to give information, but it is not effective”, A 15-year-old boy also said, “There is teaching about drinking alcohol too. Nevertheless, I think this class is not interesting”. The expected intervention is that volunteers come up with an idea. A 16-year-old declared, “I would like the community to teach more about the disadvantages of drinking alcohol. If they know that drinking has drawbacks, they can reduce drinking”. A parent suggested, “If there is a control measure for being stricter to selling alcohol, for example, not selling it to children under 18 years old”. A group of teachers maintained, “We want the government to get involved in providing knowledge and assistance community by supporting the budget and staff”.

## Discussion

The current study illustrated the extreme lifetime prevalence and the last 12-month prevalence of alcohol consumption among adolescents in a Thai rural community. In comparison with the 2018 Global Status Report on Alcohol and Health indicating that the prevalence of current alcohol consumption among young Thai adults accounted for 27.3%^1^, the present study demonstrated that the prevalence was substantially high (53.0%). Furthermore, a 2018 study in this rural area revealed that the last 12-month prevalence of alcohol consumption among adolescents was 36.8%^4^. Thus, the prevalence of alcohol consumption has risen dramatically over three years. Most related reports demonstrated that young males tended to consume alcohol more than females^1,6^. Unexpectedly, the last 12-month prevalence of alcohol consumption among males (52.1%) was comparable to that among females (53.6%) in the present study. Additionally, after adjusting for potential confounders, we found that alcohol use within 12 months among females was relatively high. Hazardous and harmful alcohol use were assessed by AUDIT score, revealing that two-fifths of current drinkers were classified into hazardous and harmful alcohol consumption which was higher than those in the data from the Thai National Household Survey for Substance and Alcohol Use^18^.

The current study demonstrated that adolescents aged ≥ 16 years tended to consume alcohol within 12 months in comparison to those who are younger. However, the average age at initiation of alcohol consumption was 13.9 years. Moreover, approximately 60% of drinkers started alcohol use at the age of less than 15 years. These initiation ages were low compared to the NSO in Thailand, which demonstrated that the average age to start drinking among Thai people outside the municipal area was 20.5 years, and 12.5% of drinkers started consuming alcohol at the age of < 15 years^2^. This phenomenon may be explained by our qualitative data as follows: although the national legal minimum age for on-/off-premises sales of alcoholic beverages was implemented, adolescents in this remote rural area could still easily access alcohol. Retailers did not enforce the policy or recognize the age of adolescents when they purchased alcohol. Moreover, they tended to understand that parents had asked their children to buy alcohol for them. Additionally, we found that adolescents did not know the long-term risks of alcohol consumption; they only recognized short-term effects such as being drunk and decreased level of consciousness. The study conducted by Hingson et al. illustrated that people who initiate drinking before age 14 were more likely to experience alcohol dependence than those who began drinking at age 21 (adjusted hazard ratio of 1.78)^19^. In addition, the early onset of alcohol consumption was associated with substance use^4^, family violence, injuries, suicide, and sexual behaviors^5^. Our study suggested that these issues should be solved promptly. Primary prevention programs should be implemented in the community. Regarding hazardous drinkers, effective therapy may be initiated and delivered by nurses working at primary care units in this rural area^20^.

Current drinker status among adolescents in this community was associated with individual and environmental factors. A significant association was found between current smokers and alcohol consumption. This finding was in compliance with related reports in the US^21,22^ and the UK^23^ that people who decided to try smoking tended to try alcohol and vice versa^22^. From the qualitative information, the teachers believed that smoking may initiate alcohol consumption which resulted from imitating the behaviors of family members.

Both quantitative and qualitative findings indicated that adolescents having friends consuming alcohol tended to be current drinkers. Adolescents, parents, and teachers affirmed that adolescents were provided an opportunity to consume alcohol by their friends. For instance, when adolescents have a party, they themselves claim that their friends do not influence their choice of action, and when a friend allows alcohol, their decision to try alcohol lies in their own hands. This finding is also in accordance with a related study in eastern Thailand, indicating that adolescents who directly observe their friends’ drinking were more likely to be drinkers^9^. On the other hand, support from friends can help the drinkers have a chance to alleviate alcohol use^24^.

Social media use may be a potential factor affecting alcohol consumption among adolescents. Although social media use was no longer associated with current drinker status, information from the qualitative study demonstrated that adolescents could access any content via social media platforms, such as peer alcohol behavior and alcohol advertising, because of unregulated marketing on social media^25,26^. Therefore, adolescents could perceive inappropriate behaviors such as alcohol consumption as usual. Social media also facilitate pro-alcohol environments and encourage drinking^25,27^. This may be explained by social learning theory^28,29^, suggesting that adolescents realize alcohol use by observing and imitating the behavior of others. For instance, adolescents repeatedly exposed to alcohol content shared by their friends could motivate the initiation of alcohol use or increase drinking^30,31^.

The present study indicated that alcohol consumption may contribute to further problems including love relationships between adolescents and their boyfriends/girlfriends. Related studies supporting this finding demonstrated that alcohol consumption negatively affects relationships^32^. In addition, a meta‐analysis of a longitudinal study illustrated that becoming single was associated with increased consumption at follow-up^33^. Furthermore, qualitative data revealed that alcohol consumption among adolescents may be associated with financial problems and, consequently, illegal behaviors such as thievery. This finding may be explained by adolescents excessively spending money on buying alcohol to consume, resulting in financial issues. Some evidence supported that higher debt and financial strain were positively associated with alcohol use^34,35^. Other related evidence also indicated that illegal acts, including theft and acts against persons, were more likely to be higher under the influence of alcohol. Furthermore, the acute use of alcohol, alone or in combination with other drugs, was involved with the illegal acts^36^.

To date, the existing intervention that helps in solving alcohol-related problems among adolescents in this rural area is the conventional health education provided in the school. However, the qualitative study indicated that adolescents were not interested in that program. Although the national legal minimum age for on-/off-premises sales of alcoholic beverages and restrictions for on-/off-premises sales of alcoholic beverages have been established (hours, days/places, and density), adolescents aged less than 18 years are still able to purchase alcohol at stores in this community.

It is observed that alcohol consumption among adolescents in this remote rural area is still a substantial issue related to not only personal factors but also socio-ecological context, including family members, peers, and community members, as well as social and cultural norms. Our results suggest that the primary prevention programs to attenuate alcohol-related problems among adolescents in this community must be implemented across multiple levels of the socio-ecological model simultaneously^37^. A specific approach should promote attitudes, beliefs, and behaviors preventing alcohol use at the individual level^38^. Generally, knowledge about the disadvantages of alcohol use should be provided, both short- and long-term consequences on physical and mental health, such as cognitive impairment, family violence, suicide, and sexual behaviors^39^.

Another level is the relationship with family and peers. The related evidence demonstrated that parental monitoring and supervision effectively prevent the onset of alcohol consumption and misuse. For instance, parents monitor their adolescents during free time and time with friends and provide active supervision by being present during youth activities^40,41^. Effective strategies such as school strategies should be also implemented in the community^38^. The related evidence established that a routine interactive educational program encouraged adolescents to be actively engaged in forming social norms to reduce alcohol use^42,43^. Furthermore, school-based prevention can focus on self-esteem and self-efficacy, concentrating on interpersonal interactions and educating about alcohol and its harmful effects^44,45^. Besides, our findings suggested that local authorities should seriously force alcohol sellers in the community to follow the minimum age regulations for on-/off-premises sales of alcoholic beverages. The strategies at the societal level, such as establishing norms that support nonuse, are crucial for preventing alcohol use and misuse. Moreover, other social institutions, such as Buddhist temples, may play an essential role through religious beliefs that can potentially assist in preventing alcohol use^46^. Additionally, the active participation of people and community engagement will serve as potential facilitators to alleviate underage drinking^47^.

In terms of limitations, firstly, the study utilized a cross-sectional survey, which made it difficult to determine a cause-and-effect relationship between associated factors and alcohol consumption. Secondly, we carried out a quantitative study at a school in the community; therefore, a few adolescents who were outside school were not included in the present study. Thirdly, there were missing data in some variables regarding the questions in the case report form that may comprise sensitive issues; the participants may not voluntarily answer those questions. Therefore, some variables, such as GPA (missing data for 57.3%), would not be included in the multivariable analysis. Fourthly, some variables, such as personal media literacy, were not collected; therefore, unmeasured confounders may not be included in the final model. Another limitation of this study was that only adolescents in this rural area were included; thus, the study may not be generalized to the whole country but may reflect the real experience of adolescents residing in rural communities in Thailand.

## Conclusion

To sum up, we presented the situation of alcohol consumption among adolescents in a remote rural community in Thailand. The current study revealed that the prevalence of alcohol consumption was extremely high among males and females. On average, young adolescents initiate alcohol consumption at age 13. The factors affecting alcohol use included individual and environmental factors. Therefore, effective strategies should be implemented across multiple levels of the socio-ecological model simultaneously to alleviate alcohol consumption among adolescents and attenuate its complications.

## Supplementary Information

Supplementary Information.

## Data Availability

The datasets generated during and/or analyzed during the current study are not publicly available because the data set contains sensitive identifying information. Because ethical restrictions have been placed, the data sets are available from the corresponding author on reasonable request (contact Boonsub Sakboonyarat via boonsub1991@pcm.ac.th).

## Abbreviations

NSO: National Statistic Office
AUDIT: Alcohol use disorders identification test
APR: Adjusted prevalence ratio
CI: Confidence interval
SD: Standard deviation
