# Supplementary material for: Alcohol consumption and its associated factors among adolescents in a rural community in central Thailand: a mixed-methods study
Source: Sci Rep. 2022 Nov 15;12:19605. doi: 10.1038/s41598-022-24243-0 (PMC9666648; doi:10.1038/s41598-022-24243-0)
Supplement: Supplementary file 1 — Supplementary Information. [file 41598_2022_24243_MOESM1_ESM.docx]

**Alcohol Consumption and Its Associated Factors among Adolescents in a Rural Community in Central Thailand: A Mixed-Methods Study**

**Protocol for Qualitative Methods**

1. Gathering and introducing yourself
2. Giving the information about the project.
3. Inform consent process
4. Interview using the questions as follow.

**Questions for Qualitative Study**

- What do you think about the effects of alcohol use?
- What do you think about family members and parents affecting alcohol use among adolescents?
- What do you think about friends or peers affecting alcohol use among adolescents?
- What do you think about the society or community affecting alcohol use among adolescents?
- What do you think about social media affecting alcohol use among adolescents?
- How difficult is it for adolescents in the community to access alcohol?
- What do you think about the existing intervention for attenuating alcohol use among adolescents in this community? Can you suggest practical strategies for solving this problem?

1. Summary session and conclusion

----------------------------------------------------------
